# Effect of abdominal weight training with and without cough machine assistance on lung function in the patients with prolonged mechanical ventilation: a randomized trial

**DOI:** 10.1186/s13054-022-04012-1

**Published:** 2022-05-25

**Authors:** Tsai-Yi Hung, Wen-Lan Wu, Ho-Chang Kuo, Shih-Feng Liu, Chia-Ling Chang, Hui-Chuan Chang, Yuh-Chyn Tsai, Jui-Fang Liu

**Affiliations:** 1 Department of Respiratory Therapy, Kaohsiung Chang Gung Memorial Hospital, #123, Ta-Pei Road, Niaosong District, Kaohsiung, 833 Taiwan; 2 Department of Sports Medicine, Kaohsiung Medical University, Kaohsiung, Taiwan; 3 Department of Pediatrics, Kaohsiung Chang Gung Memorial Hospital, Kaohsiung, 833 Taiwan; 4 Medical Department, College of Medicine, Chang Gung University, Taoyuan, 333 Taiwan; 5 Division of Pulmonary and Critical Care Medicine, Department of Internal Medicine, Kaohsiung Chang Gung Memorial Hospital, #123, Ta-Pei Road, Niaosong District, Kaohsiung, 833 Taiwan; 6 Department of Respiratory Care, Chang Gung University of Science and Technology, Chiayi, 600 Taiwan; 7 Chronic Diseases and Health Promotion Research Center, Chang Gung University of Science and Technology, Chiayi, 600 Taiwan

**Keywords:** Prolonged mechanical ventilation, Weaning ventilator, Abdominal weight training, Cough machine

## Abstract

**Purpose:**

The patients with prolonged mechanical ventilation (PMV) have the risk of ineffective coughing and infection due to diaphragm weakness. This study aimed to explore the effect of abdominal weight training (AWT) intervention with/without cough machine (CM) assistance on lung function, respiratory muscle strength and cough ability in these patients.

**Methods:**

Forty patients with PMV were randomly assigned to three groups: AWT group (*n* = 12), AWT + CM group (*n* = 14) and control group (*n* = 14). Change of maximum inspiratory pressure (MIP), Maximum expiratory pressure (MEP) and peak cough flow (PCF) between 1 day before and 2 weeks after the intervention were compared among these three groups.

**Results:**

MIP before and after intervention in AWT group (30.50 ± 11.73 vs. 36.00 ± 10.79; *p* < 0.05) and AWT + CM group (29.8 ± 12.14 vs. 36.14 ± 10.42; *p* < 0.05) compared with control group (28.43 ± 9.74 vs 26.71 ± 10.77; *p* > 0.05) was significantly improved. MEP before and after intervention in AWT group (30.58 ± 15.19 vs. 41.50 ± 18.33; *p* < 0.05) and AWT + CM group (27.29 ± 12.76 vs 42.43 ± 16.96; *p* < 0.05) compared with control group (28.86 ± 10.25 vs. 29.57 ± 14.21; *p* > 0.05) was significantly improved. PCF before and after intervention in AWT group in AWT group (105.83 ± 16.21 vs. 114.17 ± 15.20; *p* < 0.05) and AWT + CM group (108.57 ± 18.85 vs. 131.79 ± 38.96; *p* < 0.05) compared to control group (108.57 ± 19.96 vs. 109.86 ± 17.44; *p* > 0.05) showed significant improvements. AWT + CM group had significantly greater improvements than control group in MIP and peak cough flow than control group (13.71 ± 11.28 vs 19.64 ± 29.90, *p* < 0.05).

**Conclusion:**

AWT can significantly improve lung function, respiratory muscle strength, and cough ability in the PMV patients. AWT + CM can further improve their expiratory muscle strength and cough ability.

*Trial registration* ClinicalTrials.gov registry (registration number: NCT0529538 retrospectively registered on March 3, 2022).

## Introduction

The respiratory muscles are composed of the diaphragm, the internal and external intercostal muscles, and the abdominal muscles. The diaphragm plays a key role in the process of breathing. Ventilator support can lead to diaphragmatic weakness, and animal and human biopsies have shown that short-term mechanical ventilation can lead to the early stages of diaphragmatic fiber atrophy. Levine found that in human biopsy specimens, after 18 and 69 h of mechanical ventilation, the cross-sectional areas of slow and fast muscles were reduced by 57% and 53%, respectively, resulting in significant atrophy of diaphragm muscle fibers. This may be related to increased diaphragm muscle proteolysis during periods of inactivity [1]. Measurements of the diaphragm using ultrasounds show that increases in the thickness of the diaphragm can lead to longer ventilation times, while decreases in the thickness can reduce the strength of the inspiratory muscles. These conclusions indicate that increase in the thickness of the diaphragm is related to overloading the muscle [2]. Animal experiments have confirmed that diaphragmatic myofibroblasts produce diaphragmatic dysfunction due to structural damage or atrophy, which was called ventilator induced diaphragmatic dysfunction [3]. Respiratory muscle dysfunction can also increase the chances of respiratory muscle weakness and lung infections when coupled with the prolonged use of ventilators; it can be one of the factors for failure to wean off the ventilator. Therefore, ventilator weaning training should be started as soon as possible. The difficulty and duration of the ventilator weaning process can be divided into three parts: simple, difficult, and prolonged weaning from the ventilator. Difficult and prolonged weaning will increase incidence rates of the intensive care unit, while prolonged weaning from the ventilator increases mortality rates [4]. In the integrated care system for long-term ventilator-dependent patients, the use of a ventilator for ≥ 21 consecutive days, at ≥ 6 h a day, and for 5 days uninterrupted is referred to as long-term ventilator dependence [5]. Most of this group have multiple comorbidities and are bedridden over the long term. The contraction strength of their respiratory muscles and skeletal muscles is reduced, and because they cannot cough well enough, they can suffer from the accumulation of sputum and lung collapse. Several past studies have indicated that early intervention with pulmonary rehabilitation exercises can improve respiratory muscle capacity and physical activity tolerance [6].

Pulmonary rehabilitation exercises can be divided into general exercise training (e.g., resistance training or weight-bearing/non-weight-bearing exercises for the limbs) to increase muscle strength and endurance and improve physical function, and respiratory muscle training. Lung recovery exercises for long-term ventilator-assisted patients are mostly based on respiratory muscle training, including respiratory muscle resistance training, threshold pressure training, and load training on the diaphragm and respiratory muscles [7]. Abdominal weight training is also used, in which the intra-abdominal pressure increases when the abdominal muscles contract as a result of diaphragmatic pressure differences. This stimulates the contraction of the diaphragm, which in turn strengthens the diaphragm and respiratory muscles [8]. In addition, using the cough assist machine Comfort Cough II (CC20), the positive inspiratory pressure is instantly converted into a high-flow expiratory negative pressure, which generates a strong pressure difference on the respiratory tract to simulate coughing, increase the peak flow of the cough, and effectively clear the respiratory tract if secretions and restore cough functions [9]. Fewer past studies have examined the short-term benefits of using a cough assist machine and abdominal weight training on respiratory muscles and cough function in patients with long-term ventilator use. Therefore, this study mainly explored the effect of abdominal weight training assisted by a cough assist machine on the lung function of long-term ventilator patients.

## Material and methods

### Study design and setting

This study was a randomized controlled trial with a study period of August 21, 2019 to August 13, 2020. Subjects were recruited at Kaohsiung Chang Gung Memorial Hospital, a medical center in southern Taiwan, and the study site was in a subacute respiratory care center. Subjects were accepted after evaluation to determine they met the requirements and after informed consent was obtained. The study was reviewed and approved by the hospital's Institutional Review Board (IRB: 201900885B0A3) prior to subject enrollment. This study aimed to explore the intervention of abdominal weight training (AWT) with/ without cough machine (CM) on lung function, respiratory muscle strength and cough ability in the patients with prolonged mechanical ventilation (PMV).

### Study participants

**Fig. 1:**
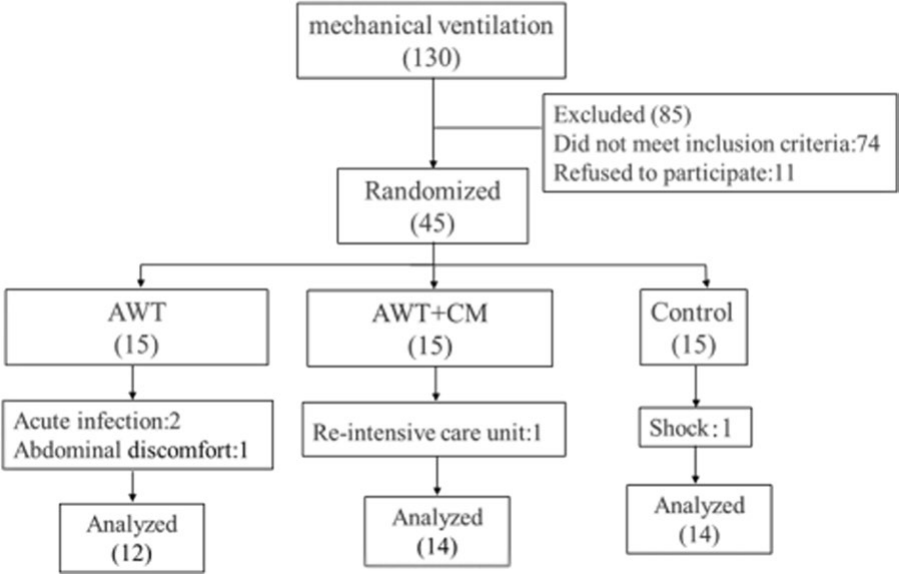
Flow chart of subject participation

Subject enrollment criteria: (1) Invasive ventilator users, (2) Hemodynamically stable, (3) Intubated endotracheal tube or tracheotomy tube, (4) Clearly conscious and cooperative, (5) Vital capacity (VC) < 10 ml/kg; Exclusion criteria: (1) Unconscious or unwilling to sign the informed consent form, (2) No spontaneous breathing, (3) Active bleeding with unstable hemodynamics, (4) Acute infection symptoms, (5) Abdominal distension, digestion problems (including nausea and vomiting), (6) Severe heart failure (ejection fraction ≤ 30%), (7) Unhealed wounds in the chest and abdomen, (8) Bullous emphysema, (9) Sensitive pneumothorax or mediastinal pneumothorax, (10) Recent history of traumatic stress, (11) Acute head and neck injury (unless the injury site is immobilized), etc. According to the order of enrollment, the participants were randomized into AWT + CM group, AWT group and control group in sequences as showed in flow diagram (Fig. 1).

### Interventions

Abdominal weight exercise training (sandbag) is maintained for 30 min; the starting weight is 1–2 kg, and the previous day's weight is maintained each day as well as adding 0.5 kg daily. Cough machine training is based on the cough assist machine Comfort Cough II (CC20), in which the inhalation and exhalation times are adjusted to 1–3 s, and the positive and negative pressure of the lower pressure 10–15 cmH_2_O is gradually increased to 30–40 cmH_2_O for the first time, 4–6 cycles/time, repeated 4–6 times, twice a day, five days a week, until the subject is weaned off the ventilator or transferred out of the ward. A modified Borg scale was used as an indicator of perceived dyspnea [10].

### Outcome Measure

The primary outcome included the change of maximum inspiratory pressure (MIP), maximum expiratory pressure (MEP), and vital capacity change between 1 day before and 2 weeks after the intervention. The secondary outcome included the outcome of weaning from the ventilator and disease severity after the intervention among three groups.

### Clinical variables

The basic information of subjects, the days of intubation, the total number of days of ventilator use, and the number of days of ventilator use in the subacute respiratory care center were collected. Respiratory function was monitored using an nSpire Haloscale Standard spirometric meter and pressure meter (Nuwass Instrumentation and Control Inc., Taiwan), which measure the parameters related to lung volume, including vital capacity (VC), tidal volume (TV), rapid shallow breathing index (RSBI), peak expiratory flow (PEF), and peak cough flow (PCF). An intensive care physiological monitoring system (Philips IntelliVue MX600), continuously measured heartbeat, respiration, blood pressure, pulse blood oxygen concentration, etc.

#### Statistical analyses

Basic descriptive statistics are presented as the mean and standard deviation. Because the distribution of the statistical population is unknown and the sample size is small, a nonparametric analysis method was used. The pretest data between groups were tested using the Kruskal–Wallis test. The Wilcoxon test was used to compare and analyze the differences between the groups before and after the test. The changes in the volume parameters, pulmonary function parameters and disease severity were tested using the Kruskal–Wallis test between groups, and then the Mann–Whitney test was used for post-hoc independent sample analysis between each group (two groups).

The number of samples accepted is estimated to have an explanatory power = 80%, moderate correlation effect size *f* = 0.25, error value *α* = 0.05. The before and after measurements of the three groups are calculated using the G*Power software, sample size error *α* = 0.05, effect size and test power were analyzed and calculated post-hoc. The SPSS 20 software was used for statistical analysis, and *p* ≤ 0.05 was used as the threshold for statistical significance.

## Results

### Flow chart of subject participation

There were 130 patients in the subacute respiratory care center during the study period, and 45 patients were eligible for admission. Among them, the participation of 5 patients (12%) was terminated, including 2 patients with acute infection and 1 patient with abdominal pain and discomfort in the abdominal weight group; 1 patient with neuromuscular disease in the AWT + CM group who was transferred to the intensive care unit; and 1 patient in the control group who went into shock (Fig. 1).

### Baseline characteristics of study participants

Forty patients with ventilator use were accepted for a randomized controlled trial and had the basic necessary conditions to be subjects in the study (Table 1). Their disease types were mostly pulmonary and respiratory diseases. The numbers of the AWT group, the AWT + CM group, and the control group were, respectively, 4 (33.3%) versus 7 (50%) versus 7 (50%), of which 6 had COPD. Clinical variables including age, gender, weight, body mass index, total duration of mechanical ventilation (days), ventilator days during respiratory care center (days), length of stay during respiratory care center (days), Charlson Comorbidity Index, APACHE II score, the percentage of tracheostomy, and primary problems at admission to respiratory care center, showed no significance among three groups.

**Table 1:** Baseline characteristics of study participants

Variable	AWT (*n* = 12) mean ± SD	AWT + CM (*n* = 14) mean ± SD	Control (*n* = 14) mean ± SD	*p*-value
Age (years)	72.08 ± 10.88	73.50 ± 10.73	67.79 ± 9.98	0.335
Sex (male/female)	6/6	8/6	8/6	0.917
Weight (kg)	60.40 ± 15.91	66.43 ± 14.48	58.89 ± 9.66	0.424
Body Mass Index (BMI)	22.29 ± 4.69	25.21 ± 5.03	22.82 ± 3.05	0.258
Total duration of MV (days)	34.83 ± 18.52	37.36 ± 15.25	40.57 ± 16.91	0.550
Ventilator days during RCC (days)	14.08 ± 12.44	19.14 ± 12.76	21.29 ± 14.55	0.381
Length of stay during RCC (days)	19.33 ± 11.59	24.21 ± 11.81	25.50 ± 12.88	0.331
Mortality prediction				
Charlson Comorbidity Index, CCI	6.83 ± 1.64	7.00 ± 2.69	6.36 ± 2 .76	0.688
APACHE II score	14.58 ± 3.66	15.21 ± 2.81	14.07 ± 2.95	0.673
Artificial airway, *n* (%)				
APACHE II score	9 (75.0)	11 (78.6)	8 (57.1)	0.420
Tracheostomy	3 (25.0)	3 (21.4)	6 (42.9)	
Primary problems at admission to RCC, *n* (%)				
Pulmonary diseases	4 (33.3)	7 (50.0)	7 (50.0)	0.687
Cardiovascular diseases	5 (41.7)	3 (21.4)	3 (21.4)	
Neuromuscular diseases	3 (25.0)	3 (21.4)	2 (14.3)	

The data are presented as mean ± standard deviation

A = Abdominal weight training, AWT group, B = cough machine assisted abdominal weight training, AWT + CM group, C = Control group

*Kruskal–Wallis measures analysis, *p* < 0.05

### Lung function, respiratory muscle strength, and coughing ability before and after intervention

**Fig. 2:**
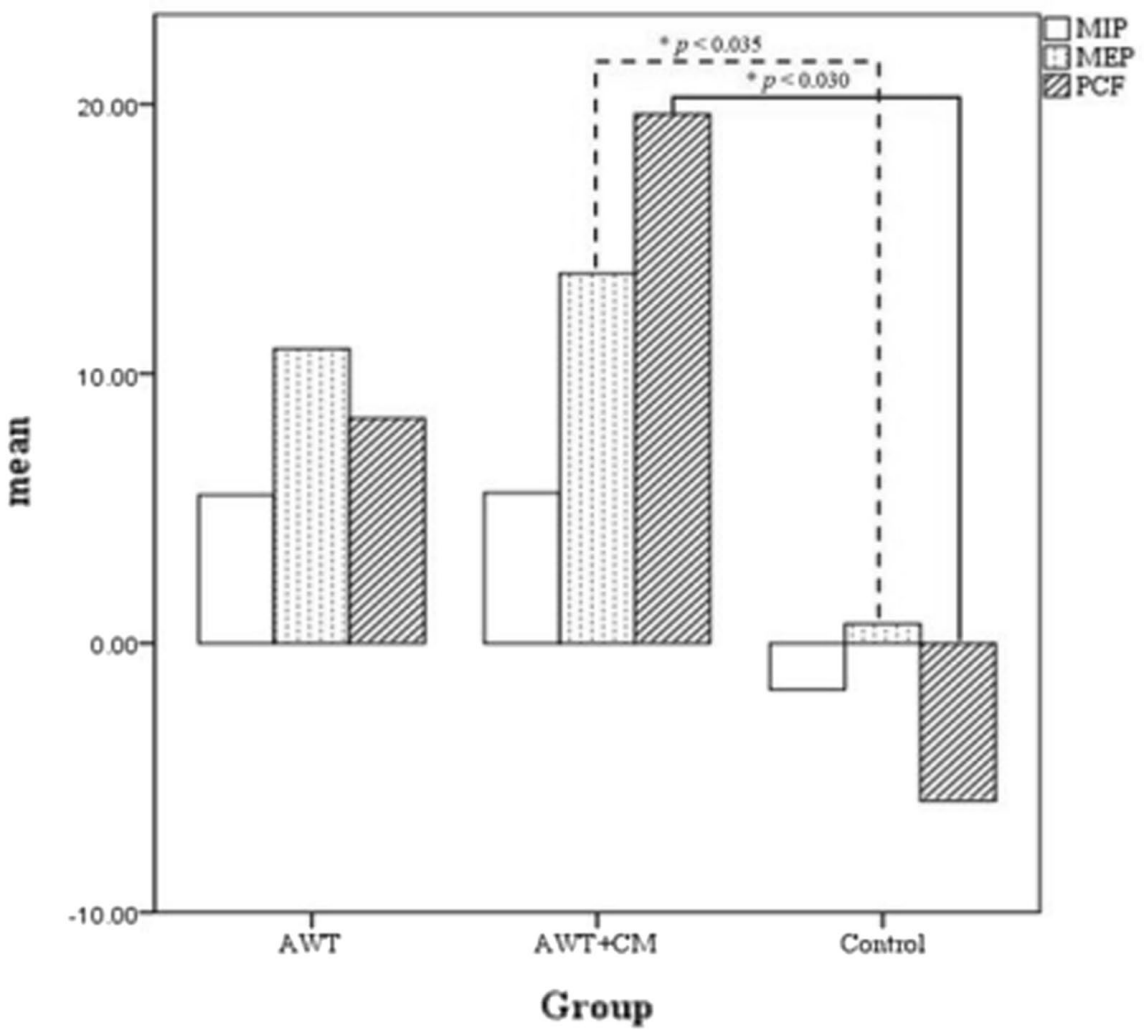
Maximal inspiratory, expiratory pressure and peak cough flow before and after intervention tween the exercise training and control groups

The effects of respiratory function parameters, respiratory muscle strength, and cough efficacy are shown in Table 2. Lung function: After training, the VC of the AWT group improved by 8% (*p* = 0.433), and the AWT + CM group improved by 27% (*p* = 0.023). The AWT group improved its RSBI by 15% (*p* = 0.034) and TV by 22% (*p* = 0.012). The AWT + CM group improved its RSBI by 18% (*p* = 0.055) and TV by 14% (*p* = 0.167). There were significant differences in both groups that received training, but there was no significant difference in the control group. Respiratory muscles: The maximum inspiratory pressure (MIP) of the AWT group improved by 18% (*p* = 0.011), and the AWT + CM group improved by 21% (*p* = 0.011); There was a 36% (*p* = 0.033) improvement in the maximum expiratory pressure (MEP) in the AWT group and a 55% improvement in the AWT + CM group (*p* = 0.001). There were significant differences in both groups that received training, but there was no significant difference in the control group. PCF in the AWT group in terms of cough efficacy: 105.83 ± 16.21 increased to 114.17 ± 15.20, an improvement of 8% (*p* = 0.011); PCF in the AWT + CM group: 108.57 ± 18.85 increased to 131.79 ± 38.96, an improvement of 21% (*p* = 0.001). There was no significant difference in the control group. Compared to the control group, the difference between the pre- and post-measured values for cough efficacy in the AWT + CM group showed significant differences in PCF (*p* = 0.030) and MEP (*p* = 0.035) (Fig. 2). There was no significant difference in the control group.

**Table 2:** Comparison of lung function and respiratory muscle strength and coughing ability of various groups

Variable	Group	Pre	Post	*p* value
		Mean ± SD	Mean ± SD	
RR (bpm)	A	24.25 ± 5.63	25.17 ± 4.06	0.503
	B	25.79 ± 5.37	23.00 ± 4.57	0.131
	C	26.14 ± 7.21	25.00 ± 6.84	0.550
RSBI	A	82.50 ± 39.87	70.25 ± 27.10	0.034*
	B	90.14 ± 36.20	70.29 ± 26.46	0.055
	C	97.79 ± 44.88	89.36 ± 38.60	0.730
TV (ml)	A	343.50 ± 132.90	404.58 ± 138.35	0.012*
	B	315.21 ± 99.81	359.79 ± 104.27	0.167
	C	278.07 ± 73.09	302.07 ± 73.14	0.258
VC (ml/kg)	A	9.95 ± 4.18	10.80 ± 3.36	0.433
	B	10.88 ± 7.19	13.86 ± 7.63	0.023*
	C	9.42 ± 6.34	9.38 ± 5.40	0.646
MIP (cmH_2_O)	A	30.50 ± 11.73	36.00 ± 10.79	0.011*
	B	29.86 ± 12.14	36.14 ± 10.42	0.011*
	C	28.43 ± 9.74	26.71 ± 10.77	0.666
MEP (cmH_2_O)	A	30.58 ± 15.19	41.50 ± 18.33	0.033*
	B	27.29 ± 12.76	42.43 ± 16.96	< 0.001*
	C	28.86 ± 10.25	29.57 ± 14.21	0.900
PEFR (L/min)	A	61.67 ± 15.72	62.92 ± 16.85	0.276
	B	57.86 ± 10.51	72.14 ± 35.72	0.080
	C	58.57 ± 16.10	61.07 ± 19.82	0.680
PCF (L/min)	A	105.83 ± 16.21	114.17 ± 15.20	0.011*
	B	108.57 ± 18.85	131.79 ± 38.96	< 0.001*
	C	108.57 ± 19.96	109.86 ± 17.44	0.753

*RR* respiratory rate, *RSBI* rapid shallow breathing index, *TV* tidal volume, *VC* vital capacity, *MIP/MIP* maximal inspiratory/expiratory pressure, *PEF* peak expiratory flow, *PCF* peak cough flow

A = Abdominal weight training, AWT group, B = cough machine assisted abdominal weight training, AWT + CM group, C = Control group

Comparison of Lung Function and respiratory muscle changes (post-study vs pre-study)

*p < 0.05 are marked as bold

### Outcome of weaning from the ventilator and disease severity after the intervention

After AWT + CM intervention, the disease severity of patients who had long-term difficulty weaning from the ventilator were compared with APACHE II: there was an improvement of 20% (*p* = 0.014), which represented a significant difference (Table 3). The overall average age was over 70 years old, and the average number of days on the ventilator was 37.73 ± 16.58. Ventilator weaning success rate: 11 in the AWT group (97.7%), 11 in the AWT + CM group (78.6%), and 11 in the control group (78.6%). Re-intubation rate: 2 in the AWT group (16.7%), 0 in the AWT + CM group (0%), and 3 in the control group (21.4%). The AWT group had fewer days of ventilator use. In the AWT + CM group, the success rate of weaning from the ventilator was 78.6%, but there were no significant differences in the number of ventilator days and re-intubation rates. Compared with the training groups, the control group had more days of ventilator use.

**Table 3:** Disease severity pre- and post-test tween the exercise training and control groups

Variable	Group	Pre	Post	*p*-value
		Mean ± SD	Mean ± SD	
APACH-II	A	14.58 (3.65)	12.17 (2.82)	0.076
	B	15.21 (2.81)	12.14 (4.09)	0.014*
	C	14.07 (2.95)	11.86 (3.70)	0.054

Within-group comparison, pre versus post

A = AWT group, B = AWT + CM group, C = control group

*p < 0.05

## Discussion

Prolonged mechanical ventilation (PMV) accounts for 15% of the subjects undergoing the ventilator weaning process, and advanced age and underlying lung disease are factors that lead to difficult and prolonged weaning [11]. Respiratory failure from pneumonia and pulmonary and respiratory diseases are the most common causes of prolonged weaning, which have an impact on the mortality during hospitalization and the success rate of extubation. A 2011 study divided the number, time, and duration of spontaneous breathing trials into simple, difficult, and prolonged ventilator weaning [12, 13]. Past literature suggests that respiratory muscle training is an effective exercise for PMV patients. Inspiratory muscle resistance training and threshold load inspiratory muscle training can increase the maximum inspiratory and expiratory pressure for severely ill patients on mechanical ventilation, COPD patients, or patients with long-term ventilator dependence. This reduces carbon dioxide accumulation in the lungs [14, 15]. The maximum inspiratory pressure (MIP) indicates the strength of the inspiratory muscle and diaphragm; the maximum expiratory pressure (MEP) indicates the strength of abdominal muscles and intercostal muscles. The maximum inspiratory pressure is related to vital capacity [16]. Ventilator weaning is based on the spontaneous breathing capacity obtained by dividing the respiratory rate by the tidal volume, which is represented by the rapid shallow breathing index (RSBI). It is a commonly used clinical reference value for predicting successful weaning from the ventilator [17]. A systematic review of inspiratory muscle training in ventilator patients included 10 studies with a total of 394 participants, in which inspiratory muscle strength and endurance, rapid shallow breathing index, and success rate of weaning were analyzed [18]. In 2018, a systematic review of 15 studies (2159 patients) on the prediction of ventilator weaning or successful extubation used RSBI as the most important predictive tool, followed by age [19]. This information can improve the rate of ventilator weaning and reduced reintubation rates. Chen proposed an inspiratory muscle training and exercise training plan, and designed 10 exercise programs for cardiorespiratory endurance, respiratory muscle strength and muscle stretching to improve lung function and reduce the degree of dyspnea [20]. There are similarities between Chen's study and this study in their conclusions and the ethnic characteristics of the subjects. With lung recovery exercise training, statistically significant differences were found in lung capacity and the maximum inspiratory and expiratory pressures (*p* < 0.05). There were no significant differences in the number of ventilator days, the success rate of ventilator weaning, and re-intubation rates. This may be due to the old ages and pulmonary and respiratory diseases of the subjects.

Cough efficacy declines with age, and researchers have compared and contrasted different methods to enhance cough efficacy (Table 4). Vinken et al. found that when the population has neuromuscular disorders, it causes a 60% reduction in MIP and a 25% reduction in VC. This is associated with chronic muscle weakness and reduced lung compliance, due to the interactions between lung volume and respiratory muscle strength. When there is only respiratory muscle weakness and no pulmonary complications, and the MIP is lower than 30% of the predicted value and VC is lower than 55% of the predicted value, then hypercapnic respiratory failure may occur [21]. Studies have confirmed that AWT + CM training increased VC (27%) and MIP improved by 21%, thus improving lung capacity and respiratory muscle strength. In 2013, researchers used the peak cough flow (PCF) as a measure of cough efficacy, and used a cough assist machine to enhance cough function for patients with lung diseases and chronic neuromuscular diseases. The normal value of PCF is 360–400 L/min; subjects with chronic diseases will have values lower than 270 L/min, and subjects with respiratory tract infection will have values of < 160 L/min, which increases the risk of ventilator dependence [22]. For subjects with cervical vertebra injuries, positive pressure is applied to the upper abdomen after inhalation to increase the maximum expiratory flow by 14%-100%, thereby achieving the effect of coughing [23]. For patients with respiratory muscle weakness, Kim showed the effect of unassisted, manually assisted following a maximum insufflation capacity maneuver, assisted by mechanical in-exsufflator, or assisted by manual thrust plus MI-E on peak cough flow. It was found that there is a significant difference on cough efficacy when assisted by manual thrust plus MI-E [24]. For the three techniques of cough assist machines, intermittent positive pressure breathing (IPPB) combined with manual thrust on the abdomen, and cough assist machine combined with manual thrust on the abdomen, there were significant differences in the visual analog scale/perceived effectiveness (6.4 vs 8.3* vs 8.5*) of IPPB combined with manual thrust on the abdomen and cough assist machine combined with manual thrust on the abdomen. However, when the expiratory flow was > 4L/s, there was no significant difference [25]. In this study, the patients were elderly and ventilator-dependent. Similar to the previously mentioned study, the PCFs were all < 160 L/min. In the AWT + CM group, the PCF increased from 108.57 ± 18.85 to 131.79 ± 38.96, an increase of 21%. Abdominal weight compression is clinically convenient, simple, and easy. It can also improve respiratory muscle strength (maximum expiratory pressure increases by 15.1 cmH_2_O, an improvement of 55%). It can improve the efficacy of coughs when combined with cough assist machines.

**Table 4:** Research on the effect on cough ability of cough assist machines and manual thrust on abdominal muscles

References	Study population	Study design	Scoring and indicators
Kim et al. 24	Patients with noninvasive ventilator-dependent neuromuscular disease	Comparisons of the effects of unassisted, manually assisted following a maximum insufflation capacity (MIC) maneuver, assisted by mechanical in-exsufflator (MI-E), or assisted by manual thrust plus MI-E on peak cough flow (PCF)	The PCF (L/min) was 95.7 (40.5) when unassisted, 155.9 (53.1) when manually assisted following an MIC maneuver, 177.2 (33.9) when assisted by MI-E, and 202.4 (46.6) when assisted by manual thrust plus MI-E* FVC (ml): 667.4 ± 313.4, improvement of 17.9%; MIP: 19.5 ± 10.2 cmH_2_O, improvement of 19.1%; MEP: 25.3 ± 19.6 cmH_2_O, improvement of 16.0%
Lacombe et al. 25	Neuromuscular patients	Comparison of three cough-augmentation techniques: insufflation by intermittent positive-pressure breathing (IPPB) combined with manually assisted coughing (MAC), mechanical insufflation-exsufflation (MI-E), and MI-E + MAC	Visual analog scale: Comfort: 6.4 vs 7.0 vs 6.6 Effectiveness: 6.4 vs 8.3*vs 8.5* All three methods are ineffective: PCF > 4 L/s, average expiratory pressure is 40 cmH_2_O
Sivasothy et al. 23	9 normal subjects, 8 patients with chronic obstructive pulmonary disease (COPD), 12 with neuromuscular diseases (including 4 subjects with respiratory muscle weakness (RMW) with scoliosis, and 8 subjects with RMW without scoliosis)	Comparing manually assisted cough and mechanical insufflation	There was no difference in peak cough flow (PCF) and cough expiratory volume (CEV) in normal subjects. PCF: Normal: 668 → 624; COPD: 370 → 245*(CEV/L, PVT/ms: 0.8, 40); scoliosis with RMW:288 → 362(CEV/L, PVT/ms:0.6, 50); RMW:104 → 248*(CEV/L, PVT/ms:0.6, 75)
Liu et al.	Long-term ventilator patients	Comparing cough reinforcement methods: unassisted, abdominal weight training (AWT), cough machine assisted abdominal weight training (AWT + CM)	In the AWT + CM group, the maximum inspiratory pressure (MIP) improved by 21%, the maximum expiratory pressure (MEP) improved by 55%, and the peak expiratory flow (PCF) improved by 21% There was a 27% improvement in VC (*p* = 0.023)

This study enrolled only a small number of subjects, which may limit the validity of inferred results. The number of subjects, the duration, and the number of centers should be increased in future studies to collect more comprehensive pulmonary function data for respiratory muscle training, including maximum voluntary ventilation (MVV), forced vital capacity (FVC), forced expiratory volume in 1 s (FEV1), peak expiratory flow rate (PEFR) and maximal mid‐expiratory flow (MMEF25-75%), thereby gaining a deeper understanding of the benefits of cough machine assisted abdominal weight training. Training should be offered earlier in the intensive care unit, and follow-ups should be conducted. Discussions should be conducted by combining diseases in the same category. In the treatment offered in clinical care and domestic research, the abdominal weight can vary between 2 and 5 kg, which is maintained between 15 and 30 min, which indicates a lack of scientific rigor and makes it difficult to explore the benefits. Future studies may more deeply examine the application of abdominal pressure.

## Conclusions

Abdominal weight exercise training improves lung function and respiratory muscle strength, and is easy to perform and easily portable. Cough machine assisted abdominal weight training can effectively improve vital capacity and improve respiratory muscle and cough functions, which can be used as a reference when selecting auxiliary training for respiratory muscles in clinical care.

## Data Availability

The datasets used and/or analysed during the current study are available from the corresponding author on reasonable request.

## Abbreviations

AWT: Abdominal weight training group
AWT + CM: Cough machine assisted abdominal weight training group
CC20: Comfort cough II
VC: Vital capacity
TV: Tidal volume
RSBI: Rapid shallow breathing index
PEF: Peak expiratory flow
PCF: Peak cough flow
MIP: Maximum inspiratory pressure
MEP: Expiratory pressure
